# Supplementary material for: Self-reports of Dutch dog owners on received professional advice, their opinions on castration and behavioural reasons for castrating male dogs
Source: PLoS One. 2020 Jun 22;15(6):e0234917. doi: 10.1371/journal.pone.0234917 (PMC7307733; doi:10.1371/journal.pone.0234917)
Supplement: S1 Appendix — For this survey-based research we determined the advice owners received from three types of dog professionals (veterinarian practitioners, behavioural therapists, behavioural trainers) and owners’ assessments of castration’s behavioural effects and this appendix lists survey items in English. (DOCX) [file pone.0234917.s001.docx]

**S1 Appendix. Questionnaire items.** For this survey-based research we determined the advice owners received from three types of dog professionals (veterinarian practitioners, behavioural therapists, behavioural trainers) and owners’ assessments of castration’s behavioural effects and this appendix lists survey items in English.

**Questions posed to owners of intact and castrated dogs:**

- What is your gender?

[female/male]

- What is your highest completed education?

[elementary school/high school/lower vocational education/intermediate vocational education/undergraduate degree or higher vocational education/master’s degree or equivalent]

- What percentage of time do you take care of the dog (for which you are filling in the questionnaire)?

[0-24%/25-49%/50-74%/75-100%]

- What is your age?

[17 or younger/18-24/25-34/35-44/45-54/55-64/65 or older]

- How satisfied are you with your dog?

[completely unsatisfied/not very satisfied/moderately satisfied/satisfied/very satisfied]

- Which breed is your dog? ‘Mix’ indicates that it is not a purebred dog.

[open]

- Is your dog a pedigree dog (with certificate)?

[yes/no]

- What is your dog’s age?

[younger than 6 months/6 to 12 months/1 to 2 years/2 to 4 years/4 to 6 years/6 to 8 years/8 to 10 years/10 to 12 years/older than 12 years]

- How old was your dog when you acquired him/her?

[6 weeks to 10 weeks/10 weeks to 18 weeks/4 months to 12 months/1 to 2 years/2 to 4 years/4 to 6 years/6 to 8 years/8 to 10 years/10 to 12 years/older than 12 years]

- What is your dog’s gender?

[female/male]

- Is your dog intact or desexed, and if desexed, was this decision made by yourself?

[my dog is intact/my dog was already desexed when I acquired it/I decided to have my dog desexed/my dog is chemically desexed (injection, non-surgical)/other or don’t know]

- To which extent do you agree with the following statements about the effect of desexing on the behaviour of male dogs in general? There is no correct or incorrect answer, please indicate your own opinion. Statements can be skipped if you do not have an opinion on them.

*Answer options for all statements were [strongly disagree/slightly disagree/neutral/slightly agree/strongly agree]*

Desexing makes male dogs calmer.

Desexing increases male dogs’ trainability.

Desexing improves male dogs’ human-directed sociality.

Desexing improves male dogs’ dog-directed sociality.

Desexing diminishes dominance behaviour in male dogs.

Desexing diminishes aggression in male dogs.

Desexing diminishes male dogs’ urine marking (urinate small amounts at various specific locations).

Desexing diminishes male dogs’ roaming behaviour (running away from home or from the yard).

Desexing diminishes mounting behaviour (mounting of other animals, humans or objects) in male dogs.

Desexing generally has a positive effect on the health of male dogs.

Desexing of male dogs is unnatural and should be done only in case of acute medical necessity.

- Advice about desexing or not desexing can be provided per your request or spontaneously. Could you indicate which of the following provided you with such advice?

*Answer options for all three professionals were [con-desexing advice/neutral advice/pro-desexing advice/no advice]*

Veterinarian

Behavioural trainer

Behavioural therapist

**Questions posed to owners of castrated dogs only:**

- To which degree were the following reasons to have your dog desexed important for your decision?

*Answer options for all reasons were [not relevant as a reason for desexing/somewhat relevant as a reason for desexing/a reason for desexing/an important reason for desexing/the main reason for desexing]*

To prevent unwanted behaviour.

To correct existing unwanted behaviour.

Due to acute medical necessity.

To reduce the chance of future unwanted behaviour.

To achieve the dog’s infertility.

To no longer be bothered by the dog being in heat.

Due to desexing being mandatory for dog day care or training courses.

- If the reason for desexing was to correct existing unwanted behaviour, which behaviours did you consider to be problematic?

*Answer options for all behaviours were [not problematic/somewhat problematic/very problematic/the main problem to be corrected by desexing]*

Urine marking

Aggression

Dominance behaviour

Hyperactivity

Indoor urination or defecation

Mounting behaviour

Roaming behaviour

Other (please clarify below)

- If the reason for desexing was to correct existing unwanted behaviour, how satisfied are you with the effect that desexing has had on the unwanted behaviour?

[completely dissatisfied/slightly dissatisfied/neutral/largely satisfied/completely satisfied]

- How old was your dog at the time of desexing?

[6 weeks to 10 weeks/10 weeks to 18 weeks/4 months to 12 months/1 to 2 years/2 to 4 years/4 to 6 years/6 to 8 years/8 to 10 years/10 to 12 years/older than 12 years/I don’t know]

- Regardless of whether or not addressing aggression was one of the reasons to desex, could you indicate if and how the prevalence of aggression changed after desexing? Aggression encompasses several behaviours such as fixating, chasing, growling, baring the teeth, snarling, snapping, barking and biting. Some of these behaviours may be shown in a context that does not reflect aggression, which does not count for answering this question. If your dog very rarely or never shows aggression and this was unaffected by desexing, please choose ‘the prevalence of aggression remained unchanged’.

[the prevalence of aggression decreased strongly/the prevalence of aggression decreased slightly/the prevalence of aggression remained unchanged/the prevalence of aggression increased slightly/the prevalence of aggression increased strongly]
