# Supplementary material for: Self-reports of Dutch dog owners on received professional advice, their opinions on castration and behavioural reasons for castrating male dogs
Source: PLoS One. 2020 Jun 22;15(6):e0234917. doi: 10.1371/journal.pone.0234917 (PMC7307733; doi:10.1371/journal.pone.0234917)
Supplement: S2 Appendix — We present additional Chi-square test output, including Chi-square values, degrees of freedom and residuals for all performed analyses. In Tables A and C we compare the frequency and the nature of the advice that was received by our complete sample of dog owners (N = 491) between different types of professionals (pairwise comparisons between veterinarian practitioners, behavioural trainers, and behavioural therapists). In Tables B and D we compare the frequency and the nature of the received advice between owners of intact dogs (N = 242) and owners of castrated dogs (N = 249), and the same two subsamples are used to compare the owners’ opinions in Table E. In Table F we compare owner-reported changes in aggression between dogs that were castrated for behavioural correction (N = 145) and dogs that were castrated for other reasons (N = 104). In Table G we compare the general ownership satisfaction of owners of intact dogs (N = 242), owners of dogs that were castrated for behavioural correction (N = 145) and owners of dogs that were castrated for other reasons (N = 104) in pairwise comparisons. In all Tables, each row represents one Chi-square test. The first column of each table contains the Chi-square value and the degrees of freedom, the last column contains the P-value. Counts represent numbers of dog owners and Chi-square residuals are between brackets and identify significant deviations from expected values (i.e. >|2|, in bold). (DOCX) [file pone.0234917.s002.docx]

**S2 Appendix. Tables containing detailed Chi-square test output.** We present additional Chi-square test output, including Chi-square values, degrees of freedom and residuals for all performed analyses. In Tables A and C we compare the frequency and the nature of the advice that was received by our complete sample of dog owners (*N*=491) between different types of professionals (pairwise comparisons between veterinarian practitioners, behavioural trainers, and behavioural therapists). In Tables B and D we compare the frequency and the nature of the received advice between owners of intact dogs (*N*=242) and owners of castrated dogs (*N*=249), and the same two subsamples are used to compare the owners’ opinions in Table E. In Table F we compare owner-reported changes in aggression between dogs that were castrated for behavioural correction (*N*=145) and dogs that were castrated for other reasons (*N*=104). In Table G we compare the general ownership satisfaction of owners of intact dogs (*N*=242), owners of dogs that were castrated for behavioural correction (*N*=145) and owners of dogs that were castrated for other reasons (*N*=104) in pairwise comparisons. In all Tables, each row represents one Chi-square test. The first column of each table contains the Chi-square value and the degrees of freedom, the last column contains the P-value. Counts represent numbers of dog owners. The Chi-square residuals are between brackets and we identify significant deviations from expected values (i.e. >│2│, in bold).

**Table A. Pairwise comparisons between different types of professionals (veterinarian practitioners, behavioural trainers, and behavioural therapists) for the frequency of their advice about castration towards 491 owners of male dogs.**

|  | Veterinarian practitioner (VP) | | | Behavioural trainer (BTr) | | | Behavioural therapist (BTh) | | |  |
| --- | --- | --- | --- | --- | --- | --- | --- | --- | --- | --- |
|  | Not advised | Advised | Total | Not advised | Advised | Total | Not advised | Advised | Total | P-value |
| VP - BTr  (χ2=53.2, df=1) | **137** (-7.3) | **347** (7.3) | 484 | **238** (7.3) | **224** (-7.3) | 462 | x | x | x | <0.001 |
| VP - BTh  (χ2=110.0, df=1) | **137** (-10.5) | **347** (10.5) | 484 | x | x | x | **287** (10.5) | **174** (-10.5) | 461 | <0.001 |
| BTr-BTh  (χ2=10.9, df=1) | x | x | x | **238** (-3.3) | **224** (3.3) | 462 | **287** (3.3) | **174** (-3.3) | 461 | <0.001 |

**Table B. Comparison between owners of intact (*N*=242) and castrated dogs (*N*=249) for the frequency of received advice about castration from different types of professionals (veterinarian practitioners, behavioural trainers, and behavioural therapists).**

|  | Owners of intact dogs | | | Owners of castrated dogs | | |  |
| --- | --- | --- | --- | --- | --- | --- | --- |
|  | Not advised | Advised | Total | Not advised | Advised | Total | P-value |
| Veterinarian  (χ2=19.8, df=1) | **90** (4.5) | **150** (-4.5) | 240 | **47** (-4.5) | **197** (4.5) | 244 | <0.001 |
| Trainer  (χ2=0.214, df=1) | 122 (0.5) | 110 (-0.5) | 232 | 116 (-0.5) | 114 (0.5) | 230 | 0.644 |
| Therapist  (χ2=0.140, df=1) | 147 (0.4) | 86 (-0.4) | 233 | 140 (-0.4) | 88 (0.4) | 228 | 0.709 |

**Table C. Pairwise comparisons between different types of professionals (veterinarian practitioners, behavioural trainers, and behavioural therapists) for the nature of their advice about castration (pro-castration, neutral, con-castration) towards 491 owners of male dogs.**

|  | Veterinarian practitioner | | | | Behavioural trainer | | | | Behavioural therapist | | | |  |
| --- | --- | --- | --- | --- | --- | --- | --- | --- | --- | --- | --- | --- | --- |
|  | Pro | Neutral | Con | Total | Pro | Neutral | Con | Total | Pro | Neutral | Con | Total | P-value |
| VP-BTr  (χ2=10.7, df=2) | **171** (2.2) | 117 (0.4) | **59**  (-3.2) | 347 | **89**  (-2.2) | 72  (-0.4) | **63**  (3.2) | 224 | x | x | x | x | 0.005 |
| VP-BTh  (χ2=36.0, df=2) | **171** (3.8) | 117 (1.4) | **59**  (-5.9) | 347 | x | x | x | x | **55**  (-3.8) | 48  (-1.4) | **71**  (5.9) | 174 | <0.001 |
| BTr-BTh  (χ2=7.14, df=2) | x | x | x | x | 89  (1.7) | 72  (1.0) | **63**  (-2.7) | 224 | 55  (-1.7) | 48  (-1.0) | **71**  (2.7) | 174 | 0.028 |

**Table D. Comparison between owners of intact (*N*=242) and castrated dogs (*N*=249) for the nature of received advice about castration (pro-castration, neutral, con-castration) from different types of professionals (veterinarian practitioners, behavioural trainers, and behavioural therapists).**

|  | Owners of intact dogs | | | | Owners of castrated dogs | | | |  |
| --- | --- | --- | --- | --- | --- | --- | --- | --- | --- |
|  | Pro | Neutral | Con | Total | Pro | Neutral | Con | Total | P-value |
| Veterinarian  (χ2=70.2, df=2) | **44** (-6.5) | 54 (0.8) | **52** (7.6) | 150 | **127** (6.5) | 63 (-0.8) | **7** (-7.6) | 197 | <0.001 |
| Trainer  (χ2=51.6, df=2) | **20** (-6.5) | 39 (1.0) | **51** (6.0) | 110 | **69** (6.5) | 33 (-1.0) | **12** (-6.0) | 114 | <0.001 |
| Therapist  (χ2=33.9, df=2) | **13** (-4.6) | 20 (-1.3) | **53** (5.5) | 86 | **42** (4.6) | 28 (1.3) | **18** (-5.5) | 88 | <0.001 |

**Table E. Comparison between owners of intact (*N*=242) and castrated dogs (*N*=249) for their opinions on castration favourably affecting nine behaviours at a population level.**

|  | Owners of intact dogs | | | | Owners of castrated dogs | | | |  |
| --- | --- | --- | --- | --- | --- | --- | --- | --- | --- |
|  | Disagree | Neutral | Agree | Total | Disagree | Neutral | Agree | Total | P-value |
| Trainability  (χ^2^=13.7, df=2) | **111** (2.9) | 85 (-1.5) | **5** (-3.0) | 201 | **61** (-2.9) | 77 (1.5) | **15** (3.0) | 153 | 0.001 |
| Mounting  (χ2=12.6, df=2) | **94** (3.5) | **90** (-2.6) | 15 (-1.4) | 199 | **43** (-3.5) | **89** (2.6) | 18 (1.4) | 150 | 0.002 |
| Aggression  (χ2=10.7, df=2) | **112** (2.3) | 80 (-0.9) | **6** (-2.9) | 198 | **67** (-2.3) | 68 (0.9) | **16** (2.9) | 151 | 0.005 |
| Sociality_human_  (χ2=8.7, df=2) | **121** (2.8) | **75** (-2.4) | 4 (-1.4) | 200 | **69** (-2.8) | **76** (2.4) | 7 (1.4) | 152 | 0.013 |
| Sociality_dog_  (χ2=8.0, df=2) | **114** (2.5) | 76 (-1.6) | 9 (-2.0) | 199 | **67** (-2.5) | 71 (1.6) | 15 (2.0) | 153 | 0.018 |
| Calm  (χ2=5.9, df=2) | **94** (2.1) | 84 (-0.8) | 23 (-1.9) | 201 | **56** (-2.1) | 72 (0.8) | 29 (1.9) | 157 | 0.052 |
| Marking  (χ2=4.27, df=2) | 94 (2.0) | 85 (-1.1) | 20 (-1.3) | 199 | 56 (-2.0) | 74 (1.1) | 22 (1.3) | 152 | 0.118 |
| Roaming  (χ2=3.43, df=2) | 65 (1.9) | 96 (-1.3) | 39 (-0.5) | 200 | 36 (-1.9) | 84 (1.3) | 33 (0.5) | 153 | 0.180 |
| Dominance  (χ2=2.78, df=2) | 105 (1.5) | 80 (-0.9) | 14 (-1.2) | 199 | 68 (-1.5) | 68 (0.9) | 16 (1.2) | 152 | 0.250 |

**Table F. Comparison between owners who castrated their dog for behavioural correction (*N*=145) and owners who castrated their dog for other reasons (*N*=104) for their reports of changes in their dog’s aggression following castration.**

|  | Owners of dogs castrated for behavioural correction | | | | Owners of dogs castrated for other reasons | | | |  |
| --- | --- | --- | --- | --- | --- | --- | --- | --- | --- |
|  | Decreased | Unchanged | Increased | Total | Decreased | Unchanged | Increased | Total | P-value |
| Aggression change after castration (χ2=16.2, df=2) | **48** (4.0) | **49** (-2.8) | 17 (-1.2) | 114 | **8** (-4.0) | **41** (2.8) | 14 (1.2) | 63 | <0.001 |

**Table G. Pairwise comparisons between owners of intact dogs (*N*=242), owners who castrated their dog for behavioural correction (*N*=145) and owners who castrated their dog for other reasons (*N*=104) for their reports of general ownership satisfaction.**

|  | Owners of intact dogs (Int) | | | Owners of dogs castrated for behavioural correction (Cor) | | | Owners of dogs castrated for other reasons (Oth) | | |  |
| --- | --- | --- | --- | --- | --- | --- | --- | --- | --- | --- |
|  | Less than very satisfied | Very satisfied | Total | Less than very satisfied | Very satisfied | Total | Less than very satisfied | Very satisfied | Total | P-value |
| Int-Cor  (χ2=10.6, df=1) | **74** (-3.3) | **167** (3.3) | 241 | **68** (3.3) | **76** (-3.3) | 144 | x | x | x | 0.001 |
| Cor-Oth  (χ2=0.00, df=1) | 74 (0.0) | 167 (0.0) | 241 | x | x | x | 32 (0.0) | 72 (0.0) | 104 | 0.991 |
| Cor-Oth  (χ2=6.79, df=1) | x | x | x | **68** (2.6) | **224** (-2.6) | 144 | **32** (-2.6) | **72** (2.6) | 104 | 0.009 |
