# Supplementary material for: Self-reports of Dutch dog owners on received professional advice, their opinions on castration and behavioural reasons for castrating male dogs
Source: PLoS One. 2020 Jun 22;15(6):e0234917. doi: 10.1371/journal.pone.0234917 (PMC7307733; doi:10.1371/journal.pone.0234917)
Supplement: Attachment — Submitted filename: Response to Reviewers 1.docx [file pone.0234917.s004.docx]

Wageningen, May 27^th^, 2020

Dear reviewer,

Thank you for helping us improve the manuscript further.

**1) Sample size**

We have added the Chi-square power calculation in lines 196-199. Please note that consequential to your second request, the maximum df in our study changed. This change was incorporated into the adaptations made to this section.

**2) Presentation of results**

We see now how the simplified tables help to improve the result presentation. The adaptation into simpler presentation of proportions and P-values has been processed throughout. We moved additional information into appendices. The Tables now indicate the P-values for each comparison separately. For instance Table 1 compares owners of castrated dogs and owners of intact dogs being advised or not for each professional in three separate Chi-square tests. The adaptations for Table 1 start at line 260 and for Table 2 at line 286. Table 3 also presents the comparisons, with P-values for each (starting at line 310) and Tables 4 and 5 were replaced by text to further facilitate ease of reading.

Sincerely,

Pascalle E.M. Roulaux MSc.

Wageningen University

Behavioural Ecology Group, Department of Animal Sciences

P.O. Box 338

6700 AH Wageningen

The Netherlands

Mr. S. Russell Clegg, Ph.D.

Academic Editor PLOS ONE

1160 Battery Street

Koshland Building East, Suite 225

San Francisco, CA 94111

United States

Wageningen, May 27^th^, 2020

Dear Mr. Russell Clegg,

Thank you for the new constructive comments by the reviewer and please allow us to explain how we dealt with these in this letter.

**1) Sample size**

We have added the Chi-square power calculation in lines 196-199. This was done in addition to our referenced argumentation on how the power was satisfactory per request of the reviewer. Please note that consequential to the reviewers second request, the maximum df in our study changed. This change was incorporated into the adaptations made to this section.

**2) Presentation of results**

We performed Chi-square tests on larger cross tables and used residuals to identify observed values that deviated from expectations, which is the common ‘post hoc’ approach for Chi-square analyses. The reviewer suggested to simplify the tables and thus improve result presentation. This has now been processed throughout. Please note that that similar results came from this directly answering if the proportion of outcomes of one factor (i.e. the ratio of counts) differs per outcome of the second factor.

So, as requested by the reviewer, the Tables now indicate the P-values for each comparison separately. For instance Table 1 compares owners of castrated dogs and owners of intact dogs being advised or not for each professional in three separate Chi-square tests. The adaptations for Table 1 start at line 260 and for Table 2 at line 286. Table 3 also presents the comparisons, with P-values for each (starting at line 310), as requested by the reviewer and Tables 4 and 5 were replaced by text to further facilitate ease of reading. We valued keeping the residuals available, but present these in S2 Appendix now, as these provide valuable information on the contribution of single counts on the overall test statistic and P-value.

With these adaptations made to our manuscript, we expect to have addressed all points raised by the reviewer.

Sincerely,

Pascalle E.M. Roulaux MSc.

Wageningen University

Behavioural Ecology Group, Department of Animal Sciences

P.O. Box 338

6700 AH Wageningen

The Netherlands
